# Exploring the biological basis of acupuncture treatment for traumatic brain injury: a review of evidence from animal models

**DOI:** 10.3389/fncel.2024.1405782

**Published:** 2024-08-07

**Authors:** Minmin Wu, Wenjing Song, Lili Teng, Jinting Li, Jiayu Liu, Hanwen Ma, Ge Zhang, Jiongliang Zhang, Qiuxin Chen

**Affiliations:** ^1^Department of Rehabilitation Medicine, Heilongjiang University of Chinese Medicine, Harbin, China; ^2^The First Affiliated Hospital of Heilongjiang University of Chinese Medicine, Harbin, China

**Keywords:** traumatic brain injury, acupuncture, fundamental mechanisms, apoptosis, animal, review

## Abstract

Traumatic brain injury (TBI) occurs when external physical forces impact the brain, potentially causing long-term issues such as post-traumatic stress disorders and cognitive and physical dysfunctions. The diverse nature of TBI pathology and treatment has led to a rapid acceleration in research on its biological mechanisms over the past decade. This surge presents challenges in assessing, managing, and predicting outcomes for TBI cases. Despite the development and testing of various therapeutic strategies aimed at mitigating neurological decline after TBI, a definitive cure for these conditions remains elusive. Recently, a growing focus has been on preclinical research investigating acupuncture as a potential treatment method for TBI sequelae. Acupuncture, being a cost-effective non-pharmacological therapy, has demonstrated promise in improving functional outcomes after brain injury. However, the precise mechanisms underlying the anticipated improvements induced by acupuncture remain poorly understood. In this study, we examined current evidence from animal studies regarding acupuncture’s efficacy in improving functional outcomes post-TBI. We also proposed potential biological mechanisms, such as glial cells (microglia astrocytes), autophagy, and apoptosis. This information will deepen our understanding of the underlying mechanisms through which acupuncture exerts its most beneficial effects post-TBI, assisting in forming new clinical strategies to maximize benefits for these patients.

## 1 Introduction

**Figure 1:**
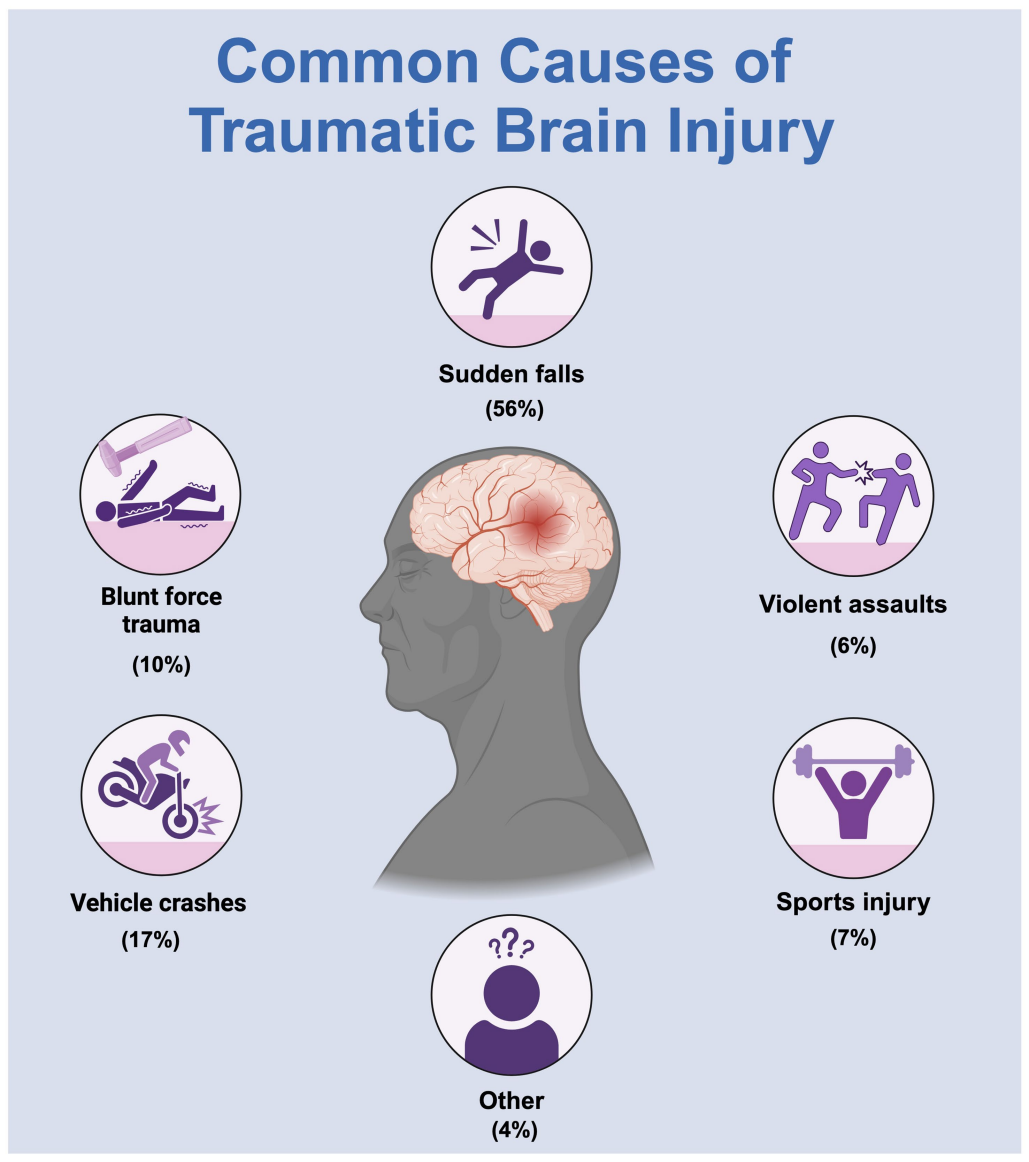
A common cause of traumatic brain injury.

Traumatic brain injury (TBI) is a brain dysfunction caused by external forces acting on the head, including impacts, blasts, sudden deceleration or acceleration, and skull penetration (Jiang et al., 2019). TBI has become a significant global public health challenge and is anticipated to persist as one of the top three causes of injury-related mortality and disability until 2030 (Maas et al., 2022; Long et al., 2024) (Figure 1). In the United States, TBI accounts for approximately 30% of all injury-related deaths annually, accounting for 61,000 deaths (Dixon, 2017). The incidence of brain injury-related emergency department visits is highest among individuals aged ≥75 years (1,682 per 100,000), followed by children aged 0–4 years (1,618 per 100,000) (Capizzi et al., 2020). Researchers are encouraged to pursue effective, safe, and inexpensive TBI treatment methods to alleviate the substantial annual economic burden of brain injuries.

Current intervention measures, such as surgery and pharmacotherapy, often yield limited success (Davis et al., 2024). Acupuncture, a traditional Chinese medical practice, is cost-effective and an important treatment method for the rehabilitation of patients with TBI. Cohort studies indicate that patients with TBI who undergo acupuncture treatment experience a lower risk of dementia (Juan et al., 2019), stroke (Shih et al., 2014), and consciousness disorders (Lin et al., 2023), along with fewer emergency and hospital visits (Shih et al., 2013), compared to those who do not receive acupuncture. Furthermore, a meta-analysis comprising four randomized controlled trials has demonstrated the effectiveness of acupuncture in alleviating symptoms among patients with TBI. These symptoms include issues related to cognition, neurology, motor functions, communication, emotions, and behavior (Wong et al., 2013).

Acupuncture improves brain function recovery and quality of life in patients with TBI; however, the biological mechanisms and potential pathways of acupuncture treatment for TBI are yet to be fully understood. Therefore, this study examined acupuncture’s potential as a treatment method for TBI by systematically reviewing foundational research on its application in animal models post-TBI. This study aimed to provide a comprehensive overview of past and current research while offering valuable suggestions for future studies.

## 2 Materials and methods

**Figure 2:**
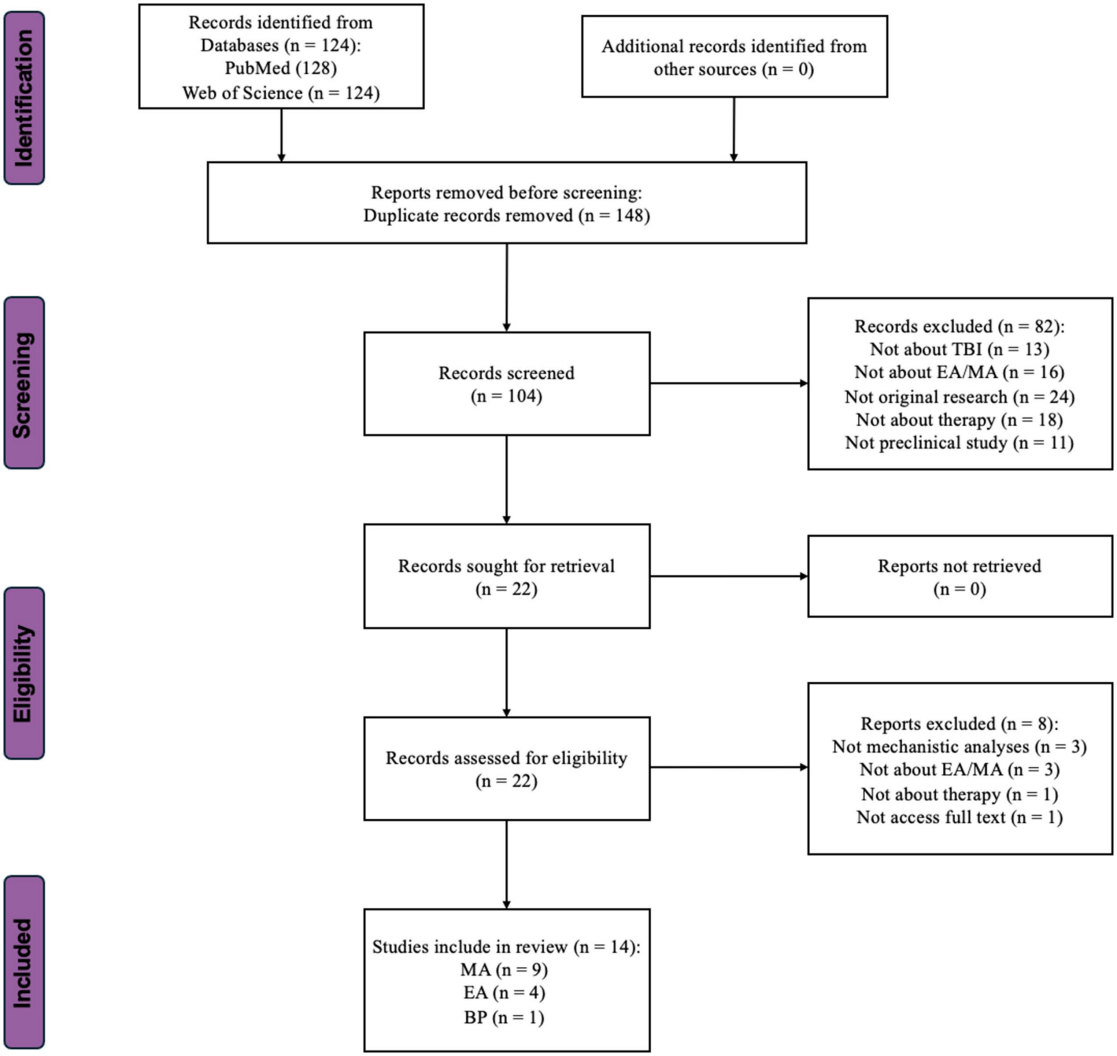
Flow-chart showing the selection/inclusion process. MA, manual acupuncture; EA, electroacupuncture; BP, bloodletting puncture; TBI, traumatic brain injury.

We conducted an extensive systematic literature search to better understand the methods and protocols employed in preclinical studies for TBI treatment. We sourced the selected studies from the PubMed and Web of Science databases. This review maintained its scientific rigor by strictly adhering to the PRISMA 2020 guidelines. Figure 2 presents a detailed explanation of the literature evaluation process.

### 2.1 Search terms: literature identification

The search included the following medical subject headings and free terms: “acupuncture” or “manual acupuncture” or “hand acupuncture” or “electroacupuncture (EA)” and “traumatic brain injury” or “TBI” or “concussion” and “animal” or “rat” or “mice”.

Only articles written in English were considered for inclusion. The query spanned all database fields, including titles, abstracts, and publications’ keywords. Full-text articles were retrieved from the selected titles, and their reference lists were searched for additional publications. Principal investigators of the included studies were contacted for additional information as needed. The search results were last updated on March 1, 2024, yielding 128 results in PubMed and 124 in Web of Science. After removing the duplicates, 104 distinct records were obtained. The results were sorted by publication date from oldest to newest. These records’ titles, authors, and publication years were exported from their respective databases and compiled into a Microsoft Excel spreadsheet for further screening.

### 2.2 Inclusion criteria: literature screening

Two review authors (WS and LT) independently screened the titles and abstracts of the initially identified studies. The search strategy yielded 104 studies, which were evaluated based on the following inclusion criteria. First, the abstract must mention TBI as the underlying potential cause of the treated condition. Second, manual acupuncture (MA) or EA must be used in the study. Third, this intervention must be used for therapeutic purposes or as a treatment method. The original study must be included, excluding other review articles and excerpts from larger studies, such as meeting abstracts and conference papers. Finally, preclinical studies must have solely used animal models to investigate certain mechanistic parameters.

A total of 82 records were excluded. Among these, 13 did not investigate the sequelae of TBI, 16 were unrelated to acupuncture, 18 used methods other than therapy, 24 were not original research, and the remaining 11 were not preclinical studies. Following abstract screening, 22 articles were selected for the next full-text assessment phase.

### 2.3 Eligibility: full-text assessment

During the full-text assessment phase, eight studies were excluded from the initial selection of 22. Among these, three studies lacked mechanistic analyses, and three involved treatments other than acupuncture. One study was not original research, which was not obvious from the abstract. Another study could not be accessed fully despite attempts to contact the author (Zhao et al., 2002). Ultimately, the literature screening resulted in 14 articles being reviewed in this study. Among them, nine independent studies employed MA (Zhang et al., 2013, 2016, 2018; Lin et al., 2018, 2020; Zhu et al., 2020; Cao et al., 2023; Zeng et al., 2023; Zhao et al., 2023), four employed EA (Chuang et al., 2013; Tang et al., 2016; Wu et al., 2023; Zhang et al., 2023), and one used Bloodletting Puncture to treat the sequelae of TBI (Liu et al., 2021).

## 3 Results

Throughout the full-text assessment, various parameters were gathered from the 14 selected articles for further analysis and comparison. The information encompassed authors, publication year, animal models, types of acupuncture, selected acupoints, specific acupuncture parameters (frequency, waveform, current intensity, duration per session, and total duration), methods of neurofunctional assessment, detection locations, pathways involved, and mechanistic changes. This information is summarized in Table 1. Additionally, information regarding the funding sources of the studies has been included in the Supplementary Table S1 (summary of study funding sources).

**Table 1:** Summary of acupuncture research on traumatic brain injury: parameters, outcomes, and mechanistic insights across animal studies.

Authors	Rat model	Treatment start time	TBI Severity	Acupuncture type	Acupoint	Acupuncture parameter	Neurological function evaluation	Test site	Involved in pathways	Final assessment time	Mechanism
Cao et al. (2023)	FFEI; SPF male Sprague–Dawley; 250 ± 20 g	12 h after modeling	Moderate (mNSS: 7–12)	MA	GV20, GV25, GV16 through GV15, LI4	15 min/d; 1–5 d	mNSS	Brain	TLR4/TRIF/MyD88	5d	CD11b/CD206 (M2 microglia) ↑ CD11b/CD86 (M1 microglia), TLR4, TRIF, MyD88↓
Wu et al. (2023)	CCI; Healthy Sprague–Dawley; 200–220 g	12 h after modeling	/	EA	LI11, LI4, GV20, CV4, ST36, KI1	1 mA; 1 Hz (dense-sparse); 15 min/d; 14 d.	mNSS, MWM, Rota rod test	Brain	AMPK/mTOR	14d	IL-10, ATP, p62, mTOR, p70S6K, 4EBP1 ↑ Neuronal apoptosis, autophagosomes, autolysosomes, ADP, AMP, LC3II, Beclin1, ATG5, ATG7, LAMP1, AMPK, TSC2 ↓
Zhang et al. (2023)	CCI; Adult male Sprague Dawley; 280–300 g	/	/	EA	GV26, PC6, GB20	2/15–100 Hz (sparse-dense); 15 min/d; 7 d.	mNSS, MWM	Brain, serum	NF-κB/COX2	7d	SOD, GSH-Px, Arg-1, IL-10 ↑ MDA, IL-6, IL-1β, TNF-α, p-NF-κB, COX2↓
Zhao et al. (2023)	FFEI; Male Sprague–Dawley; 250 ± 20 g; 8 weeks old	24 h after modeling	Moderate (mNSS: 7–12)	MA	GV20, GV26, GV16 through GV15, LI4	15 min/d; 3–14 d	mNSS	Brain (right cerebral cortex)	mTOR/ULK1	14d	p-mTOR/mTOR, p-ULK1Ser757/ULK1, p62, ↓ LC3II/I ↑
Zhang et al. (2016)	FFEI; SPF male Sprague–Dawley; 250 ± 20 g	24 h after modeling	/	MA	LI4, GV20, GV26, GV16	15 min/d; 3–14 d	–	Brain	Wnt/β-catenin	14d	Wnt3a mRNA ↑ β-catenin, sox2 protein ↑
Zhu et al. (2020)	FFEI; Adult male Sprague Dawley; 250 ± 20 g	24 h after modeling	Moderate	MA	GV15, GV16, GV20, GV26, LI4	15 min/d; 3 d	–	Brain	RhoA/ROCK2	14d	CD86, RhoA, ROCK2, IL-1β, IL-6, TNF-α ↓
Lin et al. (2018)	FFEI; SPF male Sprague–Dawley; 280 ± 20 g	12 h after modeling	mNSS: 5–8	MA	GV20, GV26, GV16, GV15, LI4	15 min/d; 3–14 d	mNSS	Brain	TLR2/4-NF-κB	14d	3 d: TLR2, TLR4, NF-κB ↑ 7 d: TLR2, TLR4, NF-κB ↓
Zhang et al. (2013)	FFEI; SPF male; 280 ± 30 g	24 h after modeling	/	MA	GV20, GV26, GV16, LI4	2 min/d; 7 d	–	Brain	–	7d	NF-200, nestin, GFAP ↑
Zhang et al. (2018)	FFEI; SPF male; 250 ± 20 g	1 h after modeling	Moderate (mNSS: 7–12)	MA	GV20, GV26, GV16, LI4	15 min/d; 3–14 d	mNSS	Brain	Notch	14d	Notch1, Hes1, Hes5 ↑
Tang et al. (2016)	FPI; Adult male Sprague Dawley; 360 ± 20 g	/	Moderate	EA	GV20, GV26, LI4, KI1	1 mA; 0.2–1 Hz (dense-dispersed); 60 min/d; 3 d.	Inclined plane	Brain	–	3d	Caspase-3, Neu-N-TUNEL, Iba1-DAPI, GFAP-DAPI, TNF-α ↓
Chuang et al. (2013)	FPI; Adult male Sprague Dawley; 280 ± 20 g	/	/	EA	GV20, GV26, LI4, KI1	1 mA; 0.2–1 Hz (dense-dispersed); 30–60 min/d; 3 d.	Run speed	Brain	–	3d	TGIF, apoptotic cell ↓
Liu et al. (2021)	CCI; Adult male Sprague Dawley; 280–320 g	/	/	Bloodletting Puncture	HTWP	15–20 μL, 2 x/d, 2 d.	mNSS, cerebral water content, MRI	Brain	MAPK	2d	MMP9, AQP4, ERK, p38 ↓
Zeng et al. (2023)	FFEI; SPF male Sprague–Dawley; 250 ± 20 g	12 h after modeling	/	MA	GV20, GV26, GV16, GV15, LI4	15 min/d; 28 d	Garcia JH Neurobehavioral Scale	Brain	–	28d	Early: astrocyte, microglia ↑ Later: astrocyte, microglia ↓
Lin et al. (2020)	FFEI; Adult male Sprague Dawley; 250 ± 20 g	24 h after modeling	Moderate	MA	GV15, GV16, GV20, GV26, LI4	15 min/d; 3 d	–	Brain	LPA-LPAR	14d	Iba1 ↓

MA, manual acupuncture; EA, electroacupuncture; CCI, controlled cortical impact; FFEI, Feeney’s freefall epidural impact; FPI, fluid percussion injury; HTWP, hand twelve Jing-well points; MRI, magnetic resonance imaging; AQP4, aquaporin 4; MMP9, matrix metalloproteinases 9; mNSS, modified neurological severity score; MWM, Morris water maze; IL, interleukin; TNF-α, tumor necrosis factor alpha; MAPK, mitogen-activated protein kinase; TLR, toll-like receptor; NF-κB, nuclear transcriptional factor-κB; NF, neurofilament proteins; GFAP, glial fibrillary acidic proteins; TGIF, transforming growth-interacting factor; SOD, superoxide dismutase; GSH-Px, glutathione peroxidase; MDA, malondialdehyde; SPF, specific pathogen free.

## 4 Mechanism of acupuncture on TBI

**Figure 3:**
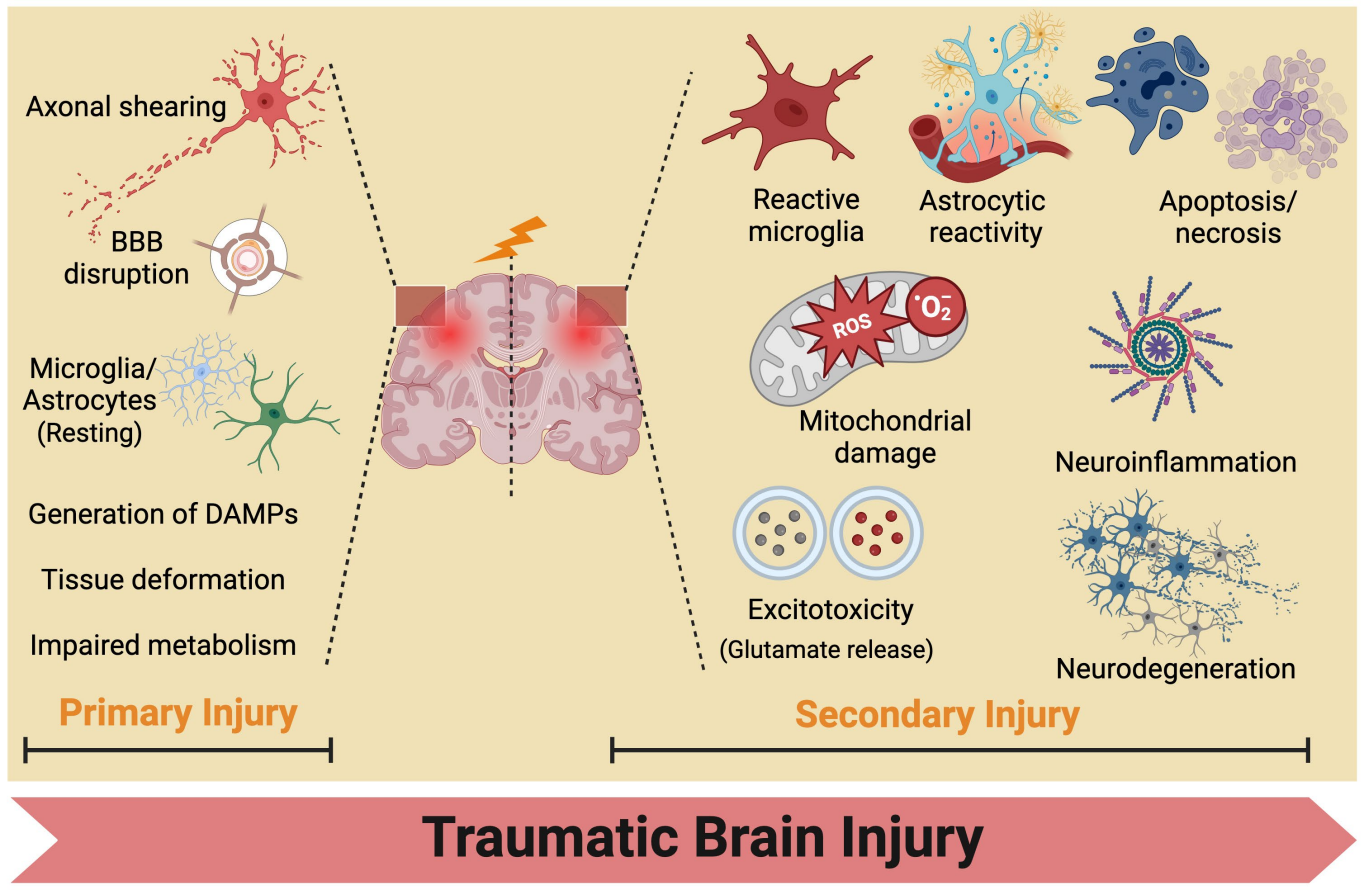
Traumatic brain injury mechanisms: from primary to secondary injury evolution. BBB, blood brain barrier.

No single theory comprehensively elucidates the pathogenesis of secondary injuries in TBI owing to its involvement in a complex cascade of reactions (Jassam et al., 2017). The progression of TBI typically entails two phases: the initial primary mechanical injury and subsequent delayed secondary injury (Kaur and Sharma, 2018). Primary mechanical injuries present as contusions, lacerations, hemorrhages, and axonal injuries, subsequently causing tissue deformation and necrosis. Secondary injuries entail a spectrum of biochemical, cellular, and molecular alterations. In the hours to days following the initial injury, neuronal and glial dysfunction, metabolic changes, neuroinflammation, and the release of signaling and inflammatory molecules from neurons, glial cells, and immune cells occur. This initiates a cascade of physiological phenomena, including activation of pro-inflammatory cytokines, disruption of the blood–brain barrier, production of reactive oxygen species, and mitochondrial dysfunction, ultimately resulting in cellular ischemia, damage, and apoptosis (Figure 3).

**Figure 4:**
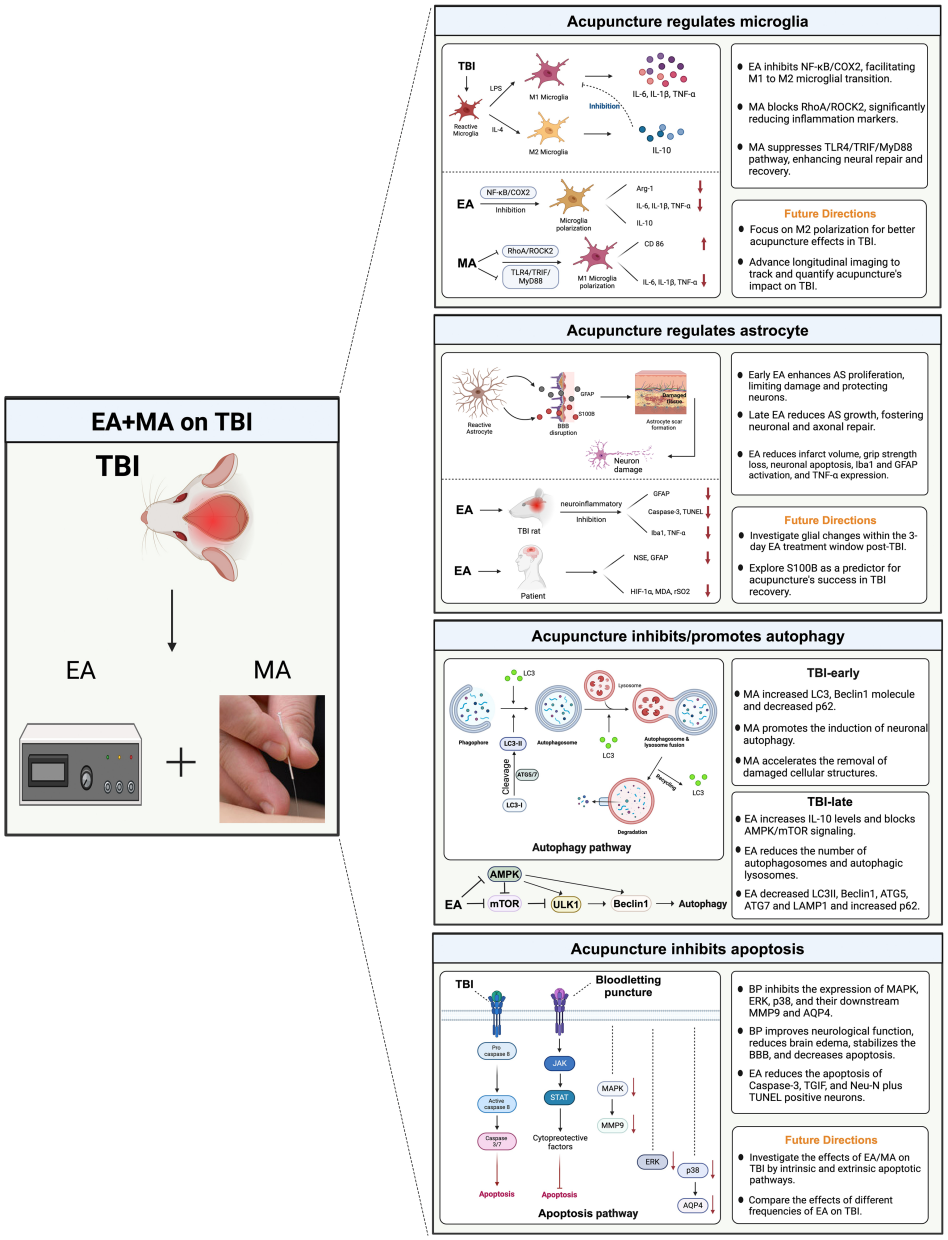
Potential mechanisms of acupuncture for traumatic brain injury. This diagram synthesizes the interactions between microglial polarization, astrocyte response, autophagic processes, and apoptotic pathways, illustrating how acupuncture potentially stabilizes cellular homeostasis and promotes neuroprotection. By influencing these key mechanisms, acupuncture aids in mitigating inflammation, supporting cellular repair, and enhancing functional recovery post-TBI. TBI, traumatic brain injury; MA, manual acupuncture; EA, electroacupuncture; AS, astrocyte.

A comprehensive understanding of these mechanisms in TBI is essential for expanding our knowledge of its pathological progression, identifying effective therapeutic targets, and formulating preventive strategies. In this context, we reviewed preclinical studies on acupuncture in animal models post-TBI to explore its effectiveness as a potential treatment approach and its mechanisms of action. Preliminary findings suggest that acupuncture may influence various biochemical pathways, including reducing neuroinflammation, promoting glial cell repair, and improving autophagy pathways, thus demonstrating its potential as a complementary treatment option (Figure 4).

### 4.1 Microglia

Microglia are involved in various homeostatic processes, including synaptic pruning, immune surveillance, and debris clearance (Chen et al., 2023). Chronic disruption of microglial homeostasis may underlie neuropsychiatric complications and neurodegenerative diseases following injury. After brain injury, microglia participate in a complex pathophysiological cascade of responses. Studies have demonstrated that TBI-associated mRNA signature and enhanced type 1 interferon signaling are apparent in cortical microglia after TBI (Witcher et al., 2021). Specifically, genes associated with synaptogenesis, calcium signaling, long-term potentiation, and dopamine signaling were suppressed in cortical neurons. Enhancements in neuronal homeostasis resulting from microglial depletion included the mitigation of post-TBI dendritic complexity, inhibition of most post-TBI transcriptional changes, interruption of neuronal connectivity and plasticity alongside functional deficits, and the mitigation of post-TBI cognitive impairments.

Under normal physiological conditions, the microglia remain quiescent. Consistent with other central nervous system injuries, microglia activation in TBI results in different phenotypes, including the pro-inflammatory M1 (neurotoxic) and anti-inflammatory M2 (neuroprotective) phenotypes (Galgano et al., 2017). M1 microglia promote the production and release of pro-inflammatory cytokines, exacerbating nerve damage, while M2 microglia promote the release of repairing and phagocytic neurotrophic factors to maintain immune system balance.

Zhang et al. (2023) confirmed that after TBI, EA regulates microglial polarization by inhibiting the NF-κB/COX2 pathway. Notably, an EA frequency of 2/100 Hz showed the best therapeutic effect. EA improved neurological and learning memory functions post-TBI and mitigated oxidative stress, evident in decreased Malondialdehyde (MDA) levels and increased superoxide dismutase and glutathione peroxidase activities. Furthermore, EA modulated microglial polarization from M1–M2, notably reducing COX2 protein expression and enhancing Arg-1 levels. TBI induced alterations in cytokine profiles within brain tissue, with EA decreasing interleukin (IL)-6, IL-1β, and tumor necrosis factor (TNF)-α levels, while increasing IL-10 levels in the brain. Similarly, MA inhibited M1 microglial polarization by modulating the RhoA/ROCK2 signaling pathway (Zhu et al., 2020). MA decreased IL-1β, IL-6, and TNF-α levels and the expression of CD86.

A previous study has recently confirmed another mechanism by which MA reduces neuroinflammation and facilitates neural repair in rats with TBI: inhibition of the TLR4/TRIF/MyD88 signaling pathway (Cao et al., 2023). Microglia predominantly transform into the M1 phenotype through binding to TLR4, TRIF, and MyD88. TLRs identify pathogen-associated molecular patterns and trigger the body’s innate immune response. TLR4 exhibited high expression in microglia, leading to increased expression of MyD88 and TRIF and activation of the pro-inflammatory pathway. MA significantly inhibited the polarization toward the M1 phenotype in microglia, indirectly fostering polarization toward the M2 phenotype, thus restoring the dynamic equilibrium of microglia. Notably, most animal model acupuncture studies on TBI focused on regulating M1 phenotypic polarization. However, future research direction should involve further investigation of the signaling pathways directly associated with M2 phenotypic polarization and the underlying mechanisms’ alterations. Additionally, conducting more longitudinal imaging studies is crucial for tracking disease progression and assessing treatment outcomes.

### 4.2 Astrocyte

S100B and glial fibrillary acidic protein (GFAP) are prominent astrocyte biomarkers. In clinical practice, serum S100B and GFAP levels are vital predictors of functional outcomes in patients with TBI (Vos et al., 2010). Specifically, elevated S100B protein levels strongly correlate with injury severity (Azar et al., 2017), poor clinical outcomes (Kleindienst et al., 2010), and higher mortality rates (Pfortmueller et al., 2016) in TBI cases. Additionally, serum GFAP levels are associated with severe functional outcomes and mortality at 6 months (Lei et al., 2015).

GFAP, an intermediate filament structural protein, is essential for maintaining cellular integrity and the resilience of the astrocyte skeleton. Its overexpression and morphological changes are the most common reactive astrocyte markers (Heimfarth et al., 2022). Systematic evaluations have shown GFAP’s utility as a biomarker for the detection of neurological disorders, including Alzheimer’s disease, Parkinson’s disease, spinal cord injury, and brain injury. Typically, under normal physiological conditions, plasma GFAP levels are very minimal. However, when the structural and functional integrity of neural cells is compromised and the permeability of the blood–brain barrier increases, it disrupts blood biomarkers, allowing GFAP to be released from tissues into the bloodstream.

Animal experiments indicated that acupuncture may exert a bidirectional regulatory effect on glial scar repair after TBI (Zeng et al., 2023). In the early stages, acupuncture promotes astrocyte proliferation (GFAP) and the formation of glial scars. This limits the extent of damage, protects neurons, and reduces neural injury. In later stages, acupuncture inhibited astrocyte proliferation (GFAP) and the hyperplasia of neural glial scars, which proved beneficial for the regeneration and repair of neurons and axons, ultimately facilitating the recovery of neural functions. Tang et al. (2016) confirmed that its anti-neuroinflammatory effects mediated the neuroprotective effects of EA in a TBI rat model. Specifically, EA treatment administered to rats for 60 min per day over 3 days significantly attenuated TBI-induced outcomes, including reduced infarct volume, grip strength loss, neuronal apoptosis (caspase-3 and TUNEL), microglia (Iba1), astrocyte (GFAP) activation, and TNF-α expression. However, future studies should characterize the changes in glial cells within the damaged brain during the 3-day EA treatment window following TBI. A randomized controlled trial revealed that 2 weeks of EA (PC 6 and GV 26) treatment improved cognitive recovery among 40 patients with mild TBI (Jia et al., 2023). The underlying mechanisms may be associated with the improved cerebral hypoxia and attenuation of cerebral damage, which included a decrease in NSE, GFAP, HIF-1α, MDA, and rSO2 levels.

S100B is a calcium-binding protein found in the cytoplasm and nucleus of astrocytes and Schwann cells. It is the most extensively studied biomarker for brain injury. S100B protein levels have been incorporated into the Scandinavian Neurotrauma Committee guidelines (Undén et al., 2013), leading to a one-third reduction in the use of computed tomography among adults in the Scandinavian region and significantly lowering healthcare costs. Despite the high sensitivity and negative predictive value of S100B in detecting traumatic intracranial hemorrhage, our review indicates that S100B has not been utilized as an efficacy indicator in animal acupuncture intervention models of TBI. A prospective study showed that serum S100B levels are elevated in patients with mild to moderate TBI; however, it did not accurately predict outcomes at 1 month (Abbasi et al., 2014). A longitudinal survey of S100B concentrations in the serum and cerebrospinal fluid (CSF) of 250 patients with TBI over 4 weeks revealed that S100B measurements could not reliably predict outcomes, and a compromised blood-CSF barrier did not affect the transfer of S100B from the CSF to serum (Kleindienst et al., 2010). Moreover, GFAP levels were more accurate in predicting non-severe and severe TBI than did S100B. However, we suggest that future basic research should attempt to use S100B as a predictor of long-term outcomes for TBI, exploring why S100B measurements fail to effectively predict outcomes due to biological or technical reasons. This could further deepen the research and may also provide scientific evidence to validate and optimize alternative treatment strategies, such as acupuncture, in promoting functional recovery from TBI.

### 4.3 Autophagy

Autophagy is an evolutionarily conserved pathway essential for maintaining cellular homeostasis. This process mediates the degradation of damaged organelles, protein aggregates, and invading pathogens via lysosome-dependent pathways (Zeng et al., 2020; Awi et al., 2021). Impaired autophagy has been associated with various pathological conditions, including traumatic brain injury, malignancy, age-related diseases, and neurodegeneration (Yin et al., 2017). There are three types of autophagy: macro-autophagy (autophagy), micro-autophagy, and chaperone-mediated autophagy. Among these, macro-autophagy is the most extensively studied type. In macro-autophagy, double-membraned organelles of the autophagosome contain cellular constituents through various steps, which are then fused to lysosomes to enable degradation. p62 is considered one of the specific substrates degraded through the autophagic process. A comprehensive review has summarized the autophagy markers used to identify TBI in the brains of trauma victims (Livieri et al., 2022). Our study aimed to validate the functional outcome of acupuncture through the autophagic pathway in animal models after TBI.

Animal studies have shown a suppression of autophagy in activated microglia and infiltrating macrophages following TBI (Hegdekar et al., 2023). This suppression contributes to an exaggerated neuroinflammatory response, persisting over weeks after injury. Researchers observed the hyperactivation of innate immune responses, including type I interferon and inflammasome pathways, which correlated with increased neuronal damage and exacerbated long-term cognitive outcomes. Their prior study revealed the suppression of macro-autophagy and autophagic fluxes in neurons post-TBI, culminating in neuronal cell death. Additionally, one study indicated that the accumulation of autophagic substrates and impaired autophagic flux independently signify suppressed autophagy post-TBI (Sarkar et al., 2014). Notably, the role of autophagy in TBI pathogenesis remains contentious. While some studies suggest a protective role for autophagy, others assert its involvement in promoting cell death. Conflicting observations regarding the beneficial and detrimental effects of autophagy post-TBI reflect a time-dependent change in autophagic flow in the injured brain. The accumulation of autophagosomes early after TBI reflects a deleterious effect in which impaired lysosomal function and autophagosome clearance lead to the accumulation of ubiquitinated proteins and protein aggregates, resulting in neuronal cell death. Autophagy may be neuroprotective when the autophagic flux is restored post-TBI. Therefore, interventions should consider the early reduction of autophagosomes and later periods when interventions can induce autophagic flux recovery, which may be neuroprotective.

Zhao et al. (2023) examined the benign regulatory effect of MA on neuronal autophagy at various time intervals of TBI through the mTOR/ULK1 signaling pathway, which reduces morphological damage in rat neurons post-TBI. They revealed that the role of autophagy in TBI largely hinges on the degree of autophagy induction and the duration of autophagy activation. Specifically, MA accelerated the elimination of damaged cellular structures by promoting the induction of neuronal autophagy for routine energy replenishment on day 3 post-TBI. In the early phase, MA elevated LC3-positive cells, LC3-I to LC3-II conversion, and proliferation of the beclin1 molecule and reduced the expression of the autophagy substrate p62 in rat neurons after TBI. Additionally, MA inhibited neuronal autophagy and curbed excessive autophagy during the late stages of TBI (days 7 and 14), consequently mitigating nerve injury. These findings suggest that autophagy is promptly activated early in TBI, inhibiting apoptosis. However, as the injury persists, autophagy is over-activated, which subsequently triggers apoptosis, inducing cell death and ultimately aggravating brain injury. Therefore, acupuncture may be a promising method for preventing the overactivation of autophagy.

An animal study revealed that EA promotes neurological recovery in rats with TBI by inhibiting excessive autophagy (Wu et al., 2023). The potential mechanism involves exerting a protective effect by increasing IL-10 levels and blocking the AMPK/mTOR signaling pathway. In their study, rats with TBI underwent daily 15-min stimulations over a continuous 14-day period through dense-disperse waves at a frequency of 1 Hz, along with a combination of acupuncture points. The combined acupoint treatment exhibited significantly superior results compared to those with individual acupoint stimulation, aligning more closely with the clinical practice of acupoint prescription. Furthermore, EA attenuated pathological damage and neuronal apoptosis in rats with TBI, leading to gradual improvement in pathological morphology, such as nuclear consolidation, fragmentation, cell swelling, neuronal necrosis, and scar tissue. In the later stages of TBI, EA enhanced the abnormal ultrastructure of brain neurons and decreased autophagosomes and autophagic lysosomes in rats with TBI. Specifically, EA reduced LC3II, Beclin1, ATG5, ATG7, and LAMP1 levels, while elevating p62 levels, thus preventing excessive autophagy in rats with TBI. EA and MA showed that neurological recovery in animals with TBI was facilitated through the autophagy pathway. Several considerations should be addressed in future studies. First, researchers should investigate the efficacy of different EA waveforms, comparing intermittent and dense-disperse waveforms, along with exploring the effectiveness of various EA frequencies, such as 1 Hz versus 100 Hz. Moreover, given that sex differences are relevant to the pathophysiological mechanisms of neurological diseases, future studies should include female and male subjects for comprehensive analysis.

### 4.4 Apoptosis

Apoptosis plays an essential role in the pathophysiology of TBI, and inhibiting it may offer potential benefits in overcoming the adverse effects of TBI and facilitating functional recovery (Unnisa et al., 2023). Apoptosis, primarily mediated by caspase-dependent mechanisms, can be initiated through the extrinsic death receptor or the intrinsic mitochondrial pathway. Within these pathways, the activation of caspase-3 serves as a pivotal marker of cell death. This activation leads to the proteolytic breakdown of DNA repair proteins, cytoskeletal proteins, and caspase-activated DNase inhibitors, ultimately culminating in morphological changes and DNA damage characteristic of apoptosis. These apoptotic processes involve the activation of cysteine-aspartic proteases, such as caspases and calpain, which are activated by the interactions of various neurochemical, cellular, and molecular pathways, including ERK, p38 MAPK, and JAK/STAT (Thapa et al., 2021). A review of studies has been conducted to delineate neuronal apoptotic pathways after TBI and their impact on the acute and chronic phases of TBI (Akamatsu and Hanafy, 2020). This study emphasizes the impact of acupuncture on apoptotic pathways, underscoring the need for future acupuncture research for TBI to investigate the intrinsic and extrinsic pathways of neuronal apoptosis thoroughly.

Apoptotic neuronal death stands as a hallmark of secondary brain injury. Studies have indicated that bloodletting at the Hand Twelve Jing-Well Points in TBI animal models can yield neuroprotective effects (Liu et al., 2021). Specifically, bloodletting puncture can suppress the expression of MAPK, ERK, p38, and their downstream molecules, MMP9 and AQP4, leading to enhanced neurological function, reduced brain edema, stabilization of the blood–brain barrier, and decreased apoptosis. In a model of brain injury induced in male rats using a fluid percussion device, researchers noted a significant increase in caspase-3-positive neurons and Neu-N plus TUNEL-positive neurons around the lesion site 72 h post-injury (Tang et al., 2016). EA treatment significantly reduced neuronal apoptosis.

Moreover, TGIF is purportedly implicated in the apoptotic signaling pathway (Wee et al., 2015). Chuang et al. (2013) compared the functional effects of 30 and 60 min of EA on rats with TBI. The results revealed that 60 min of EA intervention effectively reduced the number of apoptotic cells in the damaged cortex of rats with TBI. It also reduced the number of TGIF-positive neuronal cells, decreased the infarction volume, increased local blood flow, and ultimately improved functional outcomes in rats with TBI. These findings suggest that EA treatment mitigates the worsening of symptoms following TBI, potentially by inhibiting TGIF-mediated apoptosis in secondary brain injury post-TBI.

## 5 Limitations and future directions of research

Given the scope of the present study, several key factors warrant acknowledgment: First, bridging the translational gap between animal models and human clinical applications is challenging. Our analysis relied on animal experiments, rendering the direct translation of therapeutic mechanistic findings to acupuncture treatment in humans, particularly in patients with TBI, somewhat challenging. Second, the specificity of the acupuncture parameters in animal experiments poses challenges. We distinguished between EA and MA; however, we did not consider variations in acupoint selection. Additionally, the treatment frequency and stimulation parameters (such as the amplitude, frequency, duration, and timing of stimulation sessions) were not extensively discussed. Future studies must compare the therapeutic effects of different stimulation locations and parameters. Third, research should broaden to include comparative studies examining the efficacy of acupuncture versus other non-pharmacological interventions. This approach would contextualize acupuncture within a broader spectrum of rehabilitative treatments for TBI. Notably, all included studies were conducted in China, which may influence acupuncture methods owing to variations in researchers’ practices. Future studies in other countries should investigate the effects of acupuncture in animal models of TBI. Fourth, there are methodological limitations related to the design and reporting of the included studies. Many studies did not report critical aspects such as blinding, randomization, and power analysis. The lack of blinding may introduce bias, as researchers could consciously or unconsciously influence the results. Randomization is essential to avoid selection bias and ensure that the treatment groups are comparable. Power analysis is necessary to determine the appropriate sample size to detect a statistically significant effect, thereby increasing the reliability of the findings. Future research should adhere to the highest standards of preclinical and translational study design, including rigorous blinding, randomization, and power analysis, to enhance the validity and reproducibility of the results. Fifth, there is an inconsistency and lack of clarity in the descriptions of the Modified Neurological Severity Score (mNSS) assessments in most of the studies included in our systematic review. For example, articles by Chuang et al. (2013), Zhang et al. (2013, 2016, 2023), Tang et al. (2016), Lin et al. (2020), Zhu et al. (2020), Liu et al. (2021), Wu et al. (2023), and Zeng et al. (2023) do not describe the initial severity of TBI in animals in their methods sections. Additionally, studies by Zhang et al. (2013, 2016), Tang et al. (2016), and Zhu et al. (2020) do not report the changes in mNSS scores following acupuncture intervention in their results sections. This lack of detailed methodology can lead to variability in the application and interpretation of mNSS, affecting the comparability and reliability of the results. Furthermore, the absence of mNSS results poses significant limitations. Although our review focuses on the mechanisms of acupuncture in TBI animal models, the mNSS is a crucial measure of neurological function. The absence of mNSS results hinders the accurate assessment of the functional outcomes of acupuncture interventions. Therefore, future studies should clearly discuss and standardize mNSS assessment methods. Finally, integrating advanced imaging and neurophysiological techniques could offer deeper insights into the real-time effects of acupuncture on brain activity and connectivity in TBI models. This approach could pave the way for personalized acupuncture therapies tailored to the specific needs and injury conditions of patients with TBI. Additionally, leveraging transcriptomics techniques would enable a more comprehensive analysis of expressed factors and biological mechanisms underlying acupuncture treatment. By addressing these directions, research can offer reliable, evidence-based insights, thereby advancing the clinical application of acupuncture in treating TBI. Ultimately, this contributes to improved treatment outcomes and enhances the quality of life of patients.

Based on the current study, we suggest additional key areas to consider for future research on TBI with acupuncture. These mechanisms involve the gut flora, dysregulation of calcium signaling, mitochondrial dysfunction and oxidative stress, and pathological alterations in Tau proteins (Fesharaki-Zadeh and Datta, 2024). Alterations in the gut flora can significantly affect brain function by modulating immune responses, producing neuroactive compounds, and affecting the integrity of the gut barrier (Zhang et al., 2022; Gu et al., 2024). Acupuncture may improve neurological recovery after TBI by modulating the gut flora composition. A deeper exploration of this role may not only broaden our understanding of the neuromodulatory effects of acupuncture but also open new avenues for acupuncture in regulating the gut flora and optimizing gut-brain health in the treatment of TBI. The prevalence of calcium signaling dysregulation in TBI may be reduced using acupuncture by modulating the flow of calcium ions in neurons and their regulatory mechanisms. For example, acupuncture treatment may reduce abnormal intracellular calcium accumulation by stabilizing cell membranes, modulating impaired calcium channel function, or activating calcium pumps. Mitochondrial dysfunction and oxidative stress are key factors in the injury cascade following TBI, involving diminished energy metabolism, cell death, and increased inflammatory responses (Chang et al., 2022; Pandya et al., 2023; van Hameren et al., 2023). Acupuncture may reduce the burden on mitochondria by modulating intracellular calcium ion levels, thereby decreasing the generation of reactive oxygen species. Acupuncture may strengthen the resistance of brain cells to oxidative stress by activating the antioxidant enzyme system and reducing the release of inflammatory cytokines. These improvements not only help to protect nerve cells from further damage but may also facilitate the recovery of neurological function after TBI (Cheng et al., 2012). Pathological alterations in Tau proteins, such as hyperphosphorylation and misfolding, are key factors in neurological impairment and cognitive decline after TBI (Katsumoto et al., 2019; Shin et al., 2021; Brett et al., 2022). Acupuncture treatment may reduce pathological phosphorylation of Tau by modulating intracellular signaling pathways in neuronal cells, inhibiting overactivated kinases, or activating specific phosphatases. Therefore, future experimental models and human studies should explore in depth the specific mechanism of action of acupuncture on pathological changes in Tau and how this traditional medical technique can be effectively utilized to treat or prevent TBI-induced neurodegenerative diseases (Liu, 2023).

## 6 Conclusion

In this review, we examined animal model studies that underscore the multifaceted role of acupuncture in TBI rehabilitation. We described the intricate biological mechanisms through which acupuncture exerts its therapeutic effects. These findings underscore acupuncture’s potential as a multifaceted therapeutic intervention for promoting functional recovery in animal models of TBI. Acupuncture achieves this by modulating microglial cell activation, astrocyte function, autophagic processes, and apoptotic pathways. Future studies should further elucidate these mechanisms in humans, facilitating the integration of acupuncture into holistic TBI rehabilitation programs. The intersection of acupuncture and modern neuroscience provides a unique perspective on TBI recovery’s intricate and multifactorial nature and offers promising, more effective, and personalized treatment strategies.

## Data availability statement

The original contributions presented in the study are included in the article/Supplementary material, further inquiries can be directed to the corresponding author.

## Author contributions

MW: Data curation, Investigation, Writing – original draft, Writing – review & editing. WS: Data curation, Methodology, Writing – review & editing. LT: Data curation, Investigation, Methodology, Writing – review & editing. JLi: Data curation, Investigation, Methodology, Writing – review & editing. JLiu: Data curation, Investigation, Methodology, Writing – review & editing. HM: Data curation, Formal analysis, Methodology, Writing – review & editing. GZ: Investigation, Methodology, Writing – review & editing. JZ: Data curation, Formal analysis, Software, Writing – review & editing. QC: Conceptualization, Data curation, Funding acquisition, Supervision, Validation, Writing – original draft, Writing – review & editing.
