# Supplementary material for: Exploring the biological basis of acupuncture treatment for traumatic brain injury: a review of evidence from animal models
Source: Front Cell Neurosci. 2024 Aug 7;18:1405782. doi: 10.3389/fncel.2024.1405782 (PMC11335542; doi:10.3389/fncel.2024.1405782)
Supplement: Supplementary file 1 [file Table_1.DOCX]

**Supplementary Table 1: Summary of Study Funding Sources**

| **Authors** | **Funding Source** | **Type of Funding** | **Declaration of conflicting interests** |
| --- | --- | --- | --- |
| Cao et al. (2023) | National Natural Science Foundation of China (82174483, 81873362, 81704156 and 81574066) and the Natural Science Foundation of Guangdong Province (2021A1515011219, 2021A1515110146 and 2017A030310024). | Government | No conflicts of interest declared |
| Wu et al. (2023) | National Natural Science Foundation of China Youth Program (No.81904310); Natural Science Basic Research Program of Shaanxi Province (No.2021JQ-729); National Natural Science Foundation of China (81971049, 81671097); National Natural Science Foundation of China (82074503). | Government | No conflicts of interest declared |
| Zhang et al. (2023) | Youth Fund of the National Natural Science Foundation of China (81704155). | Government | No conflicts of interest declared |
| Zhao et al. (2023) | National Natural Science Foundation of China (81873362 and 82174483); Natural Science Foundation of Guangdong Province (2114050002002). | Government | No conflicts of interest declared |
| Zhang et al. (2016) | Project of the NationalNatural Science Foundation of China (No. 81273827), theProject of Science and Technology of Guangdong (No.2011B031800284), the Project of Scientific cultivation andinnovation foundation of Jinan University (No. 21615427) andthe Science and Technology Program of Guangdong, China (No. 2010GN-E00221). | Government | No conflicts of interest declared |
| Zhu et al. (2020) | National Natural Science Foundation of China (81704156), Natural Science Foundation of Guang-dong Province (2016A030310093 and 2017A030310024), Administration of Traditional Chinese Medicine of Guangdong Province, China (20161067 and 20181070), and the Fundamental Research Funds for the Central Universities, China (21616318). | Government | No conflicts of interest declared |
| Lin et al. (2018) | Natural Science Foundation of China (81574066), the Fundamental Research Funds for the Central Universities, China (21615427) and the Foundation of Guangdong Province Traditional Chinese Medicine Scientific Research Project (20151184) | Government | No conflicts of interest declared |
| Zhang et al. (2013) | Project of Natural Science Foundation of Guangdong (9151063201000028) and Project of Science and Technology of Guangdong (2011B031800284). | Government | / |
| Zhang et al. (2018) | National Natural Science Foundation of China (81273827), the Project of Science and Technology of Guangdong, China (2011B031800284) and the Science and Technology Program of Guangzhou, China (2010GN-E00221). | Government | / |
| Tang et al. (2016) | Chi-Mei Medical Center (CMFHR10315). | Institutional | No conflicts of interest declared |
| Chuang et al. (2013) | Chi-Mei Medical Center (CMFHR10108). | Institutional | No conflicts of interest declared |
| Liu et al. (2021) | National Natural Science Foundation of China (81873369, 81704146, 81273868 and 81330088), the Tianjin Municipal Bureau of Labor and Social Security (2018015). | Government | No conflicts of interest declared |
| Zeng et al. (2023) | / | / | / |
| Lin et al. (2020) | / | / | / |
